# Comparison of Myopic Progression and Quality of Life Wearing Either DIMs Lenses or Single-Vision Myopia Correcting Spectacles

**DOI:** 10.1155/joph/9959251

**Published:** 2025-02-12

**Authors:** Xiaoying Li, Wei Ma, Yutong Song, Maurice Yap, Longqian Liu

**Affiliations:** ^1^Department of Ophthalmology, West China Hospital, Sichuan University, Chengdu, China; ^2^Department of Optometry and Vision Sciences, West China Hospital, Sichuan University, Chengdu, China; ^3^School of Optometry, The Hong Kong Polytechnic University, Hong Kong, China

## Abstract

**Purpose:** To assess the efficacy of the Defocus Incorporated Multiple Segments (DIMS) spectacle lens for myopia control and its impact on vision-related quality of life in Chinese children over a 1-year period.

**Methods:** This randomized double-blind prospective controlled clinical trial enrolled 176 myopic subjects aged between seven and 14 years who were randomly assigned into the DIMS group or the control single-vision (SV) group. Refractive error and axial length measurements at baseline, three-, six-, nine-, and 12-month follow-up visits were monitored. The Quality of Life Impact of Refractive Correction (QIRC) questionnaire was used to evaluate the vision-related quality of life at baseline and at 12 months postintervention.

**Results:** Of the initial cohort, a total of 151 children successfully completed the study (DIMS *n* = 72; SV *n* = 79). Baseline characteristics were similar between both groups. Average axial elongation and myopia progression after 1 year were 0.17 (95% CI 0.13–0.20) mm and −0.27 (95% CI −0.36 to −0.18) D in the DIMS group and 0.30 (95% CI 0.26–0.33) mm and −0.55 (95% CI −0.64 to −0.47) D in the SV group. The mean differences in axial elongation and myopia progression were 0.13 mm (95% CI 0.08–0.18 mm, *p* < 0.001) and −0.28 D (95% CI −0.41 to −0.15 D, *p* < 0.001) between the two groups. No significant difference in the QIRC score was found between the DIMS and SV groups (55.30 (95% CI 53.17–56.90) versus 54.20 (95% CI 51.99–56.41), *p*=0.854)).

**Conclusion:** The use of DIMS lenses in children was found to slow down myopia progression compared to SV lenses, without negatively affecting their overall quality of life.

**Trial Registration:** Clinical Trial Registry identifier: ChiCTR2000037443

## 1. Introduction

For over a century, efforts have been made to correct myopia using lenses, including undercorrection and full correction of the refractive error [1, 2]. In the latter half of the last century, researchers began to systematically investigate how specific optical modifications could influence eye growth and myopia development. Bifocal spectacles were first used for myopia control [3]. With advancements in technology and the evolution of theories about myopia, more optical strategies were introduced to manage myopia progression, such as contact lenses and orthokeratology [4, 5], multifocal soft contact lenses [6], and peripheral defocus spectacle lenses [7, 8]. Using optical strategies to control or mitigate myopia progression, rather than merely correcting its symptoms with lenses, represents a significant shift in the way myopia is managed.

Early attempts at controlling myopia progression focused on two primary goals: reducing accommodative stress by using plus lenses or bifocals and discouraging further eye growth by undercorrecting manifest myopia [3, 9, 10]. While these methods showed some promise, the results were not consistent, and the underlying mechanisms remained unclear [9, 11–14].

The introduction of animal models of myopia provided researchers with valuable tools to study how visual signals could lead to abnormal eye growth [15–18]. Specific visual information carried by retinal defocus seems to regulate the growth and refractive status of the eye [19]. An early landmark experiment shed light on the compensation for lens-imposed defocus [20], as well as in other experiments [21–24]. Subsequent studies have shown a prominent role for peripheral retinal refractive state in myopia development [25–27]. These discoveries inspired the design and production of lenses that produce set levels of peripheral defocus whilst correcting central vision appropriately [28–30].

One early attempt evaluated 3 lens designs, all producing varying amounts of positive relative peripheral power from the central clear zone [31]. However, the 3 lens designs did not influence myopia progression. The lens design that was reported to achieve significant myopia control is the defocus incorporated multiple segments (DIMS) lens [8]. This design features a central zone with a diameter of 9.4 mm that provides clear distance vision, while the peripheral annular area, measuring 33 mm in diameter, induces myopic defocus through 396 microlens segments with a +3.5 D addition [8, 32].

Several studies have reported the myopia control effects of DIMS lenses [8, 33, 34], but only one randomized controlled trial (RCT) has been included, with refractive range limited to −5.00 D to −1.00 D [8]. In light of the impressive outcomes reported, there was a keen interest to see if the myopia control outcomes of the DIMS lens reported could be replicated in a real-world setting for children with a broader refractive range. Additionally, considering that DIMS lenses are designed to control myopia in children, the impact of myopia management on vision-related quality of life (VR-QoL) should be considered when making clinical decisions. The success of any myopia management strategy ultimately depends on the child's satisfaction with their visual status during treatment and their compliance with the prescribed lens wear [35]. To our knowledge, few studies have yet compared the impact of DIMS and SV lenses on VR-QoL. We conducted an RCT of the DIMS lens (Hoya MiyoSmart) in a hospital-based eye clinic setting in a large city in west China to investigate the effectiveness of DIMS lens in slowing myopia progression in children across a broader range of refractive errors and to assess its impact on VR-QoL using validated questionnaires.

## 2. Methods

### 2.1. Study Design

This study is a 1-year prospective, randomized, double-blind clinical trial. Subjects were randomly assigned to the DIMS group and SV group. The changes in spherical equivalent refraction (SER) and axial length (AL) of the two groups for 1 year were compared to assess the myopia control effect. The Quality of Life Impact of Refractive Correction (QIRC) score was used to evaluate VR-QoL. The study was approved by the Ethics Committee of West China Hospital of Sichuan University (No. 06 of 2020). All procedures followed the tenets of the Declaration of Helsinki. Parents or legal guardians signed written informed consent before child participation.

### 2.2. Subjects

Subjects were recruited at the West China Hospital of Sichuan University between January 2021 and March 2022. They were recruited by approaching parents of children undergoing vision examinations in the adolescent myopia clinics and inquiring whether they were interested in participating in a myopia control clinical trial. After providing detailed explanations of the purpose and procedures of the trial to parents and children and answering their questions, those who wished to join the trial were entered into a list. One investigator (LXY) then assessed whether the children met the inclusion criteria. The inclusion criteria were as follows:

1. Age 6∼15 years
2. Spherical refractive error of −8.00∼0.00 D
3. Astigmatism ≤ 1.50 D
4. Anisometropia ≤ 2.00 D
5. Best-corrected distant visual acuity ≤ 0.0 LogMAR
6. Willingness to accept random allocation and wear spectacle lenses as required

Children were excluded if they had strabismus, any type of binocular vision abnormality, or eye-related disease.

The success rate of subject recruitment was approximately 5 in every 10 people approached.

### 2.3. Sample Size

The previous 2-year study detected a 0.50 D (SD 0.70 D) difference between the two groups in myopia progression [8]. Since this current study was conducted for only one year, to detect potentially smaller differences with medium effect size (Cohen's *d* = 0.5), we calculated that a minimum of 62 subjects per group would be required based on a test power of 0.8 and a significance level of 0.05 (two-tailed). Considering an expected dropout rate of approximately 20%, the target sample size for each group was set at 75 participants.

### 2.4. Study Procedure

#### 2.4.1. Randomization and Masking

The study used randomization and masking techniques. A statistician independent of the trial prepared a random number table, which determined the allocation of subjects to either the DIMS or SV group based on their screening order. Odd-numbered subjects were assigned to the DIMS group, while even-numbered subjects were allocated to the SV group. To maintain masking, all lens labels were removed before distribution, making it difficult for both subjects and examiners (MW, SYT) to distinguish between SV and DIMS lenses. Subjects were guided by a designated investigator (LXY) to the examination, ensuring that the examiners did not come into close contact with the lenses.

#### 2.4.2. Measurements

The primary outcome variables were the 1-year change in subjective cycloplegic refraction and AL of both groups. Assessments were conducted at baseline and 3, 6, 9, and 12 months after wearing the lenses. Subjective refractions were carried out after the instillation of 0.5% tropicamide plus 0.5% phenylephrine hydrochloride (Mydrin -P), with one drop in each eye instilled 10 min apart, a total of 4 drops. The investigator (LXY) instilled eyedrops for subjects. Absence of pupillary light reflex was considered a proxy for “complete” cycloplegia. The masked investigators (MW, SYT) performed the subjective refraction according to the principle of “maximum plus to maximum visual acuity.” The AL was measured by the masked investigator (SYT) with an IOL Master 700 (Carl Zeiss, Oberkochen, Germany). The first five ALs with a difference of ≤ 0.02 mm and a signal-to-noise ratio higher than 5 were recorded and their average was used for data analysis.

The QIRC questionnaire adapted for use in Chinese by Xu et al. showed favorable repeatability and validity [36, 37]. The QIRC questionnaire was considered the most comprehensive and well-developed patient-reported outcome measure (PROM) for assessing VR-QoL in refractive correction [38]. Previous studies have shown that the QIRC can discriminate the effect in VR-QoL between modes of refractive correction, supporting the rationality of choosing it as an assessment tool [39, 40]. This modified QIRC consisted of a total of 20 questions. Each question had five response levels that were assigned scores based on Rasch weighting principles [37]. Its interval scoring properties allow for distinguishing varying levels of VR-QoL impact, providing statistically interpretable results. The true quality-of-life score was calculated by summing the item scores and then dividing this value by the number of answered items. Participants filled out the QIRC at both baseline and after a 12-month follow-up period, with assistance from their guardians if needed.

#### 2.4.3. Lenses Replacement

The final distance prescription was determined by the cycloplegic subjective refraction. Lenses were replaced when the SER changed by more than 0.50 D or the corrected visual acuity was worse than 0.18 LogMAR.

### 2.5. Statistical Analysis

Data from the right eye were used for analysis using SPSS V.27.0 (IBM Corp. Armonk, NY, USA). Firstly, normality analysis and homogeneity of variance (Levene's test) test were performed on the data. The *t*-test analyzed the means of continuous variables that conform to the normal distribution while the Wilcoxon rank test compared those that do not conform to the normal distribution. The data were described as the mean, 95% CI, median, and interquartile range (IQR). The *χ*^2^ test was used to compare the categorical data. Pearson correlation was used to assess the association of each factor (age, baseline AL, and baseline refraction) with myopia progression.

Repeated measures analysis of variance (ANOVA) was used to compare the differences in myopia progression and AL growth between the two groups. The changes in SER and AL at each visit of the two groups were analyzed by unpaired *t*-test or Mann–Whitney U test. Multiple tests were performed using Bonferroni corrections. A two-sided *p* value < 0.05 was considered statistically significant.

## 3. Results

### 3.1. Baseline Characteristics

**Figure 1:**
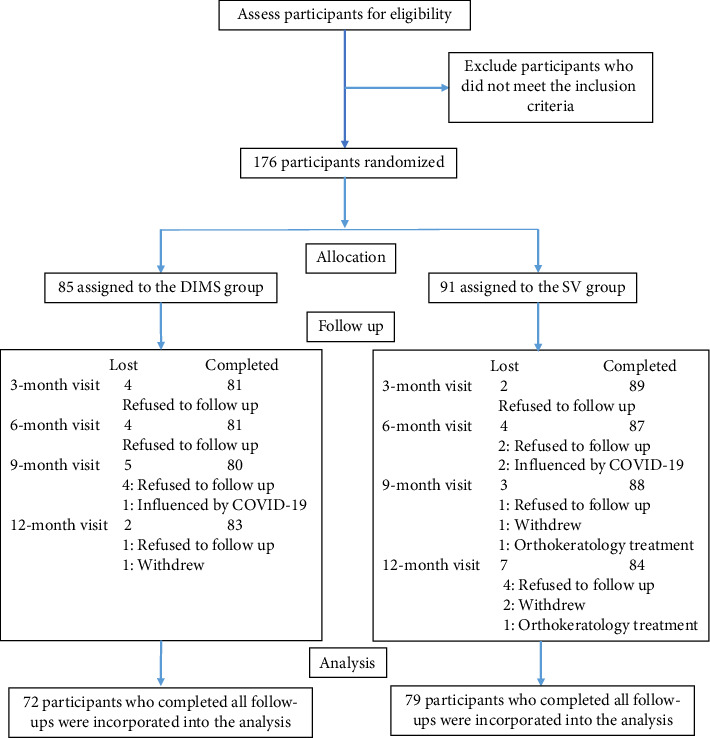
The flowchart of the study shows the number of subjects who completed the assessments at each follow-up visit, as well as those who were lost to follow-up and the reasons for their attrition. The subjects who refused to follow up often cited heavy study pressure as the reason. They mentioned that they did not have the time to visit the hospital eye clinic for the assessments.

A total of 176 myopic children were enrolled in this study and randomly assigned to the DIMS group and the SV group (85 vs. 91). Seventy-two children in the DIMS and 79 children in the SV group completed all the follow-up visits. The completion of subjects at each follow-up visit throughout the trial period is shown in Figure 1. Only data from 151 children who completed each follow-up visit were included in the analysis. Ocular characteristics were similar in the two groups (Table 1). The SER ranged from 0 D to −6.75 D in the DIMS group and 0 D to −6.50 D in the SV group, with average values of −3.16 (95% CI −3.54 to −2.78) D and −3.13 (95% CI −3.49 to −2.77) D, respectively (*p*=0.910). The mean ALs in the DIMS and SV groups at baseline showed no significant difference of 24.82 (95% CI 24.62–25.03) mm versus 25.02 (95% CI 24.79–25.26) mm, *p*=0.204. Other variables, such as gender, age, total QIRC score, flat, and steep keratometry, did not show any significant difference.

### 3.2. The Changes in Myopia Progression

**Figure 2:**
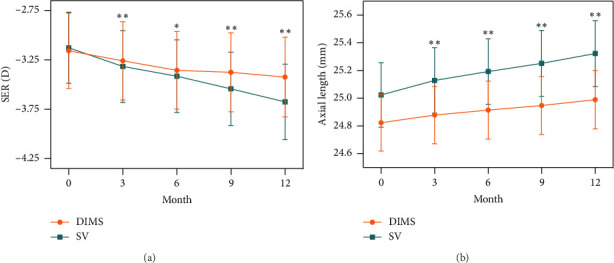
One-year spherical equivalent refraction (a) and axial length (b) changes in the DIMS group and SV group. ⁣^∗^*p* < 0.05, ⁣^∗∗^*p* < 0.001.

To assess the difference between groups in myopia progression, we conducted a repeated-measures ANOVA, which revealed a statistically significant interaction effect (time × group, *p*  <  0.001). Analyzing simple effects, we found that at each visit, subjects in the DIMS group experienced significantly slower myopia progression compared to those in the SV group (*p* < 0.05), as shown in Figure 2(a).

The average myopia progression was −0.27 (95% CI −0.36 to −0.18) D in the DIMS group and −0.55 (95% CI -0.64 to −0.47) D in the SV group for 1 year. The mean differences in myopia progression were −0.28 D (95% CI -0.41 to −0.15 D, *p* < 0.001) between the DIMS and SV groups. At each follow-up, the rate of myopia progression was lower in the DIMS group than in the SV group (Table 2).

**Figure 3:**
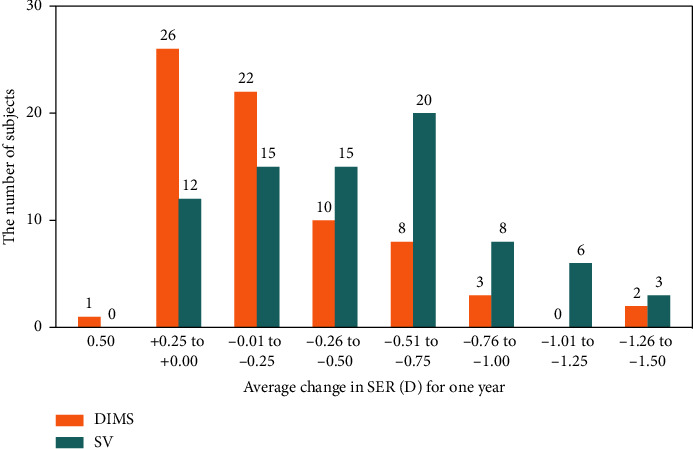
Frequency distribution graph of the change in spherical equivalent refraction after wearing DIMS lenses and SV lenses for 1 year.

Children wearing DIMS lenses exhibited a higher proportion of myopia nonprogression than those wearing SV lenses (38% VS 15%, *p*=0.002). The proportion of myopia progression no more than 0.25 D in the DIMS group was 68%, while that in the SV group was only 34% (*p* < 0.001). The proportion of myopia progression over 0.75 D was 7% in the DIMS group and 22% (*p*=0.011) in the SV group (Figure 3).

### 3.3. The Changes in Axial Elongation

The interaction effect is also statistically significant in axial elongation (*p* < 0.001). The axial elongation in the DIMS group is significantly less than that in the SV group (within-subjects *p* < 0.001, between subjects *p* < 0.001) (Figure 2(b)).

The average axial elongation was 0.17 (95% CI 0.13–0.20) mm in the DIMS group and 0.30 (95% CI 0.26–0.33) mm in the SV group for 1 year. The mean difference in axial elongation between the DIMS and SV groups was 0.13 mm (95% CI 0.08–0.18 mm, *p* < 0.001).  Table 3 presents the mean axial elongation values at 3, 6, 9, and 12 months for each group, as well as the mean differences between the two groups. After wearing DIMS lenses for 1 year, 8 children had no axial elongation or even regressed, while all children wearing SV lenses had axial elongation.

### 3.4. Factors Associated With Myopia Progression and Axial Elongation

**Figure 4:**
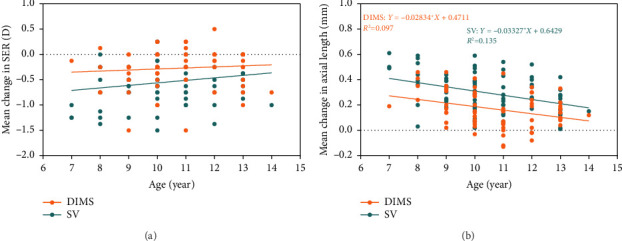
The correlation of myopia progression (a) and axial elongation (b) with baseline age in the DIMS group and SV groups.

The average change in SER for 1 year was not associated with age, baseline SER, and baseline AL in either the DIMS or SV groups, despite older age and children with lower myopia showing less myopia progression (Figure 4(a)).

There was a negative correlation between the axial elongation and age in both the DIMS (*r* = −0.291, *p*=0.013) and SV (*r* = −0.363, <0.001) groups, which indicated that older children with myopia experienced slower axial growth (Figure 4(b)). However, there was no correlation between axial growth and baseline SER and baseline AL.

**Figure 5:**
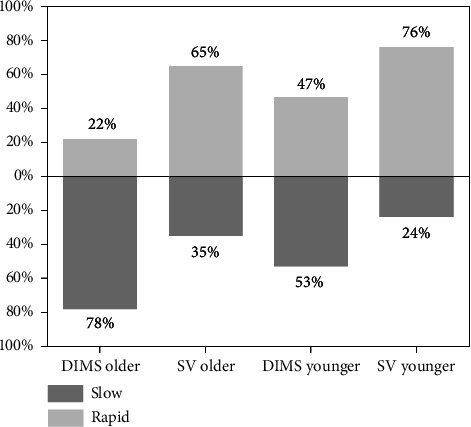
The percentage of rapid and slow progressors in the DIMS group and SV groups among younger and older subjects.

To reveal the further impact of age on axial elongation, children were divided into younger subjects (7–10) and older subjects (11–14) according to the median age (10 years old) to explore the percentage of rapid and slow progressors. Children whose axial elongation exceeds 0.2 mm were considered to have rapid progression. The results showed (Figure 5) that among younger subjects, the proportion of rapid axial elongation was 47% and 76% in the DIMS group and SV group, while among older subjects, the proportion of rapid axial elongation was 22% and 65%, respectively. In the DIMS group, the proportion of rapid progression among older subjects was significantly lower than among younger subjects (*p*=0.023), whereas no difference was observed in the SV group (*p*=0.293).

### 3.5. VR-QoL of Wearing Glasses for 1 Year

The higher the score, the better the VR-QoL. After wearing glasses for 1 year, there was no statistically significant difference in the total QIRC score between the DIMS and SV groups (55.30 (95% CI 53.17–56.90) versus 54.20 (95% CI 51.99–56.41), *p*=0.854). Similarly, no significant differences were found between the two groups for any specific question (Table 4). We also compared baseline and 1-year QIRC scores in the DIMS group. The results indicated that there were no statistically significant differences in the total score or individual item scores before and after wearing lenses.

## 4. Discussion

This study demonstrates that myopic children wearing DIMS lenses have significantly less myopia progression compared to children wearing SV lenses. The DIMS lenses were initially designed by Lam et al. [8] based on the myopic defocus mechanism for myopia control. Their RCT demonstrated that wearing DIMS lenses resulted in 52% less in the progression of myopia compared to wearing SV lenses. However, subsequent retrospective studies on DIMS lenses and other related research have indicated that myopia control effectiveness is modest, with myopia control efficacy ranging from 21% to 40% [33, 34, 41]. This could be attributed to the inclusion of children with higher degrees of myopia in these studies, as children with higher degrees of myopia tend to experience faster progression of myopia [42]. In the present study, the percentage reduction in myopia progression (51%), including highly myopic children, is similar to the findings of Lam et al. [8], thereby supporting the positive myopia control effect of wearing DIMS lenses. Its myopia control effect is comparable to that of orthokeratology lenses [43, 44], although it is inferior to low-concentration atropine [45–47]. However, recent studies on low concentrations (0.01%) of atropine have indicated unsatisfactory outcomes in terms of myopia control [48, 49].

In the present study, the effect sizes (Cohen's d) for changes in SER and AL are −0.71 and 0.90, respectively, which suggests that the differences between the two groups are meaningful. The effect sizes are the quantification of differences and tell us how large this difference actually is. A larger effect size means a larger difference, that is, the intervention has a greater impact. Our results show that wearing DIMS lenses slows down myopia progression compared with wearing SV lenses, not only does it make sense statistically, but it also makes sense in practical applications.

In comparison to the 1-year treatment efficacy reported by Lam et al. [8]—which showed a slowing of myopia progression by 0.38 D and a reduction in axial elongation by 0.21 mm—the present study observed a myopia progression delay of −0.28 D and a decrease in axial elongation by 0.13 mm. When analyzing a subset of 114 subjects in the present study who met the same inclusion criteria as Lam et al., we observed a weaker effect (0.26 D and 0.14 mm). It is worth noting that the present study was conducted during the COVID-19 pandemic, which likely exposed subjects to higher levels of myopia risk factors, such as reduced outdoor time and increased screen time [50]. These additional challenges may have contributed to the smaller effects observed in our study.

Previous studies found that in children with high myopia, the myopia control effect of DIMS lenses is not significant [33, 34]. It was suggested that the limited myopic defocus provided by the DIMS lenses might not meet the threshold required for myopia control in highly myopic eyes, as the effectiveness of myopic defocus is, to a certain extent, dependent on dosage [7, 51]. Regardless of lens type, lenses that corrected higher amounts of central myopia resulted in a more hyperopic relative peripheral defocus shift than lower-powered lenses of the same type [52], which means that the amount of myopia defocus provided by a higher-diopter DIMS lens will decrease. However, we found the initial degree of myopia is not related to the myopia progression or the axial elongation when using DIMS spectacles. Although our study also did not find statistically significant differences in myopia progression between the DIMS and SV groups among children with high myopia, those wearing DIMS lenses still demonstrated a slower myopia progression compared to those wearing SV lenses. Currently, DIMS lenses only provide a fixed myopia defocus of 3.5 D. Perhaps a larger myopic defocus will be more effective for children with high myopia. The myopia control effect of combining DIMS lenses with low-concentration atropine is notably superior to that of using DIMS lenses or low-concentration atropine alone [41, 53]. Hence, children with high myopia might benefit from considering this combined approach for improved myopia control. The study included only a small number of subjects (*n* = 23) with high myopia, and larger and more targeted trials with substantial sample sizes are required to assess the effectiveness of DIMS lenses in controlling myopia progression in individuals with high myopia.

In the DIMS group, we did not observe a significantly positive correlation between myopia progression and age (*r* = 0.113, *p*=0.346), which is inconsistent with the significant findings of previous studies [8, 33]. The discrepancy could be attributed to the fact that myopia progression was really low in the DIMS group (67% of them had a progression of −0.25 D to +0.25 D). Also, the changes in SER are not continuous variables, as diopters are recorded in 0.25 D increments. In that way, the myopia progression tends to be more correlated to the myopia control effect by DIMS, instead of to the age. Our scatterplots (Figure 4(b)) indicated that axial elongation negatively correlated with age, suggesting that older children experienced slower axial elongation. Furthermore, age has a greater impact on the DIMS group compared to the SV group. In the DIMS group, the proportion of rapid progression decreased from 47% in younger subjects to 22% in older subjects, whereas in the SV group, the proportion of rapid progression remained relatively stable across both younger and older subjects. Older subjects may potentially benefit more from wearing DIMS lenses, as the proportion of rapid myopia progression decreased from 65% in the SV group to 22% in the DIMS group among older subjects. Previous studies have indicated that DIMS lenses are more effective in children with baseline hyperopic relative peripheral refraction (RPR) compared to those with baseline myopic RPR [54]. It has been observed that children aged 8 and 9 years tend to have significantly more myopic RPR than older children, which may result in a reduced myopia control effect in this age group [54].

We observed that children wearing DIMS lenses experienced significantly less myopia progression in the last 6 months (−0.07 D) compared to the first 6 months (−0.20 D), which aligns with findings from previous studies [8, 53]. This could be attributed to the gradual reduction of accommodative lag after wearing DIMS lenses [55], and it was noticed that children with higher accommodative lag tended to exhibit more myopia progression [56].

Apart from myopia control, it is also important to investigate the broader impact of the DIMS lenses on VR-QoL which identified several key domains, namely, activity limitation, convenience, mobility, emotional well-being, comfort symptoms, and social and economic issues [57, 58]. Previous research showed that spectacle and/or contact lens wearers reported discomfort and inconvenience from wearing their correction, difficulty performing activities, and dysfunctionality, resulting in introversion, embarrassment, and self-consciousness [35, 58]. The impact of VR-QoL is an important factor for practitioners to consider when deciding on the ideal myopia management strategy for children. In recent years, PROM instruments (validated questionnaire) have increasingly gained importance as an outcome measure [38]. The results of the present study showed no difference in the overall QIRC score between the two groups, indicating that DIMS lenses may provide a similar VR-QoL to that of SV lenses. Although the study suggested that DIMS lenses may reduce contrast sensitivity [59], this change does not appear to negatively impact VR-QoL. The potential benefits of DIMS lenses in slowing myopia progression and reducing the risk of myopia-related pathologies in children may provide long-term VR-QoL improvements. It is important to note that the QIRC questionnaire was developed based on adults with refractive correction by spectacles, contact lenses, and refractive surgery and was not specifically designed for myopia control lenses [37]. It may lack the sensitivity needed to detect differences between groups in children's reported VR-QoL associated with DIMS and SV lenses. Researchers face the challenge of selecting the most suitable PROM that is both high-quality and sensitive enough to align with the research protocol. Currently, there is no PROM instrument specifically tailored for myopia control, making the QIRC questionnaire an acceptable choice for this study.

Previous studies have indicated that VR-QoL is higher with children wearing multifocal soft contact lenses or orthokeratology compared to spectacles [40, 60, 61]. The Low-Concentration Atropine for Myopia Progression (LAMP) study found no differences in VR-QoL between the placebo and the atropine groups [62]. How the VR-QoL of DIMS spectacle lenses compare with other myopia management methods is unknown. Future research could assess the VR-QoL with different myopia control methods, providing clinical practitioners valuable insights when deciding on optimal myopia management strategies for children.

Our study had several limitations. In common with the Lam et al. [8] study, more observant and experienced clinicians could identify the DIMS lens by the image profile, thus breaching the masking. However, there was neither the incentive nor time in the busy eye clinic for our two examiners to do so. The children could theoretically do the same, but the chance of this happening was remote. Another limitation is the duration of the wear regime. Although we found statistically significant effects after just 12 months of DIMS lens wear, the differences in SER and AL between groups were relatively small. A longer study would enable us to comment on the cumulative effects of the DIMS lens.

In summary, we were able to replicate the findings reported by Lam et al. [8] regarding the efficacy of the DIMS lens in controlling myopia in our hospital outpatient eye clinic setting for children across a broader range of refractive errors. Additionally, for the first time, we compared the impact of wearing DIMS lenses versus SV lenses on VR-QoL, revealing no significant difference.

## 5. Conclusion

The DIMS lens was found to slow down myopia progression among myopic children. Wearing DIMS lenses had no negative effects on the VR-QoL.

## Figures and Tables

**Table 1:** Baseline demographic data of completed subjects.

	DIMS (*n* = 72)	SV (*n* = 79)	*p* value	Effect size
Age (years)	10.69 (10.33∼11.06)	10.38 (10.00∼10.76)	0.237	−0.19 (−0.51∼0.13)
Gender, male/female	43/30	44/35	0.744	—
SER (D)	−3.16 (−3.54∼−2.78)	−3.13 (−3.49∼−2.77)	0.910	0.02 (−0.30∼0.34)
Axial length (mm)	24.82 (24.62∼25.03)	25.02 (24.79∼25.26)	0.204	0.21 (−0.11∼0.53)
Flat keratometry (D)	42.66 (42.30∼43.02)	42.64 (42.33∼42.95)	0.925	−0.02 (−0.34∼0.30)
Steep keratometry (D)	43.98 (43.60∼44.36)	43.85 (43.50∼44.20)	0.617	−0.08 (−0.40∼0.24)
Total QIRC score	54.29 (52.31∼56.28)	51.84 (49.82∼53.85)	0.087	−0.28 (−0.60∼0.04)

*Note:* The data are represented by mean (95% CI). All variables were analyzed by unpaired t-test except for gender which was analyzed by *χ*^2^ test. The effect sizes were represented as Cohen's *d*, the same follow as.

Abbreviations: D, diopters; DIMS, defocus incorporated multiple segments lens; QIRC, Quality of Life Impact of Refractive Correction; SER, spherical equivalent refraction; SV, single vision lens.

**Table 2:** Average change in spherical equivalent refraction for 1 year.

	Myopia progression (D)		Mean difference (95% CI)	*p* value	Effect size
	DIMS	SV			
3-month	−0.10 (−0.15∼−0.05)	−0.19 (−0.23∼−0.15)	−0.09 (−0.15∼−0.02)	0.002	−0.42 (−0.75∼−0.10)
6-month	−0.20 (−0.26∼−0.14)	−0.29 (−0.35∼−0.23)	−0.09 (−0.17∼−0.01)	0.029	−0.36 (−0.68∼−0.03)
9-month	−0.22 (−0.30∼−0.14)	−0.41 (−0.49∼−0.34)	−0.20 (−0.30∼−0.09)	< 0.001	−0.60 (−0.92∼−0.27)
12-month	−0.27 (−0.36∼−0.18)	−0.55 (−0.64∼−0.47)	−0.28 (−0.41∼−0.15)	< 0.001	−0.71 (−1.04∼−0.38)

*Note:* The data are represented by mean (95% CI). The changes in SER were analyzed by the Mann–Whitney U test.

**Table 3:** Average elongation in axial length for 1 year.

	Axial elongation (mm)		Mean difference (95% CI)	*p* value	Effect size
	DIMS	SV			
3-month	0.06 (0.04∼0.07)	0.10 (0.09∼0.12)	0.05 (0.03∼0.07)	< 0.001	0.81 (0.48∼1.14)
6-month	0.09 (0.07∼0.11)	0.17 (0.15∼0.19)	0.08 (0.05∼0.11)	< 0.001	0.86 (0.53∼1.19)
9-month	0.12 (0.10∼0.15)	0.23 (0.20∼0.26)	0.10 (0.07∼0.14)	< 0.001	0.88 (0.54∼1.21)
12-month	0.17 (0.13∼0.20)	0.30 (0.26∼0.33)	0.13 (0.08∼0.18)	< 0.001	0.90 (0.56∼1.23)

*Note:* The data are represented by mean (95% CI). The changes in AL were analyzed by unpaired *t*-test.

**Table 4:** The QIRC score of wearing DIMS and SV lenses for 1 year.

Items	DIMS				SV				*p* value	Effect size
	Mean	95% CI	Median	IQR (p_25_ to p_75_)	Mean	95% CI	Median	IQR (p_25_ to p_75_)		
Total score	55.03	53.17∼56.90	55.56	48.68 to 61.61	54.20	51.99∼56.41	56.15	47.02 to 63.09	0.854	−0.09 (−0.41∼0.23)
Total score (Q1 to Q13)	50.40	48.53∼52.28	51.15	44.10 to 57.10	49.63	47.57∼51.69	49.97	43.52 to 57.10	0.712	−0.09 (−0.41∼0.23)
Total score (Q14 to Q20)	63.53	60.88∼66.19	64.51	56.62 to 74.49	61.86	58.80∼64.92	64.40	51.98 to 74.49	0.662	−0.13 (−0.45∼0.19)
1. How much difficulty do you have driving in glare conditions?	51.37	48.57∼54.17	60.51	45.06 to 60.51	48.82	45.84∼51.80	60.51	29.61 to 60.51	0.263	−0.20 (−0.52∼0.12)
2. During the past month, how often have you experienced your eyes feeling tired or strained?	52.66	49.83∼55.50	49.66	49.66 to 65.11	53.37	50.46∼56.28	49.66	49.66 to 65.11	0.662	0.06 (−0.27∼0.38)
3. How much trouble is not being able to use off-the-shelf (nonprescription) sunglasses?	47.35	44.42∼50.28	56.71	41.26 to 56.71	46.08	43.05∼49.10	56.71	28.51 to 56.71	0.621	−0.10 (−0.42∼0.22)
4. How much trouble is having to think about your spectacles or contact lenses before doing things; e.g., traveling, sport, going swimming?	49.78	46.81∼52.75	45.92	45.92–61.37	51.21	48.25∼54.17	61.37	45.92 to 61.37	0.449	0.11 (0.21∼0.44)
5. How much trouble is not being able to see when you wake up; e.g., to go to the bathroom, look after a baby, see alarm clock?	46.87	43.73∼50.02	43.87	28.42 to 59.32	47.12	44.11∼50.14	43.87	28.42 to 59.32	0.924	0.02 (0.30∼0.34)
6. How much trouble is not being able to see when you are on the beach or swimming in the sea or pool, because you do these activities without spectacles or contact lenses?	53.62	50.70∼56.55	63.92	48.48 to 63.92	53.07	50.00∼56.13	63.92	33.03 to 63.92	0.886	−0.04 (−0.37∼0.28)
7. How much trouble are your spectacles or contact lenses when you wear them when using a gym/doing keep-fit classes/circuit training etc.?	46.68	43.87∼49.50	55.17	39.72 to 55.17	47.14	44.44∼49.84	55.17	39.72 to 55.17	0.805	0.04 (−0.29∼0.36)
8. How concerned are you about the initial and ongoing cost to buy your current spectacles and/or contact lenses?	54.24	51.31∼57.16	64.61	49.16 to 64.61	55.46	52.63∼58.30	64.61	49.16 to 64.61	0.465	0.10 (−0.23 ∼0.42)
9. How concerned are you about the cost of unscheduled maintenance of your spectacles and/or contact lenses; e.g., breakage, loss, new eye problems?	46.74	43.68∼49.80	45.18	29.73 to 60.62	47.58	44.57∼50.60	45.18	29.73 to 60.62	0.660	0.06 (−0.26∼0.39)
10. How concerned are you about having to increasingly rely on your spectacles or contact lenses since you started to wear them?	53.49	50.45∼56.53	50.01	34.56 to 65.46	52.66	49.48∼55.84	50.01	34.56 to 65.46	0.756	−0.06 (−0.39∼0.26)
11. How concerned are you about your vision being not as good as it could be?	48.12	44.99∼51.25	49.69	34.24 to 65.14	47.30	44.05∼50.55	49.69	34.24 to 65.14	0.668	−0.06 (−0.39∼0.27)
12. How concerned are you about medical complications from your spectacles and/or contact lenses?	50.00	47.17∼52.82	59.49	44.04 to 59.49	47.70	47.17∼52.82	44.04	44.04 to 59.49	0.240	−0.19 (−0.52∼0.14)
13. How concerned are you about eye protection from ultraviolet (UV) radiation?	54.86	51.85∼57.87	51.17	51.17 to 66.62	52.39	49.30∼55.48	51.17	35.72 to 66.62	0.290	−0.19 (−0.52∼0.14)
14. During the past month, how much of the time have you felt that you have looked your best?	61.12	56.98∼65.26	60.79	45.52 to 79.18	58.21	54.26∼62.16	60.79	45.52 to 79.18	0.284	−0.17 (−0.48∼0.16)
15. During the past month, how much of the time have you felt that you think others see you the way you would like them to (e.g., intelligent, sophisticated, successful, cool, etc.)?	69.06	65.51∼72.61	64.26	64.26 to 82.65	68.42	64.42∼72.42	82.65	64.26 to 82.65	0.843	−0.04 (−0.36∼0.29)
16. During the past month, how much of the time have you felt complimented/flattered?	68.69	64.46∼72.92	69.82	54.55 to 88.21	67.56	63.26∼71.85	69.82	54.55 to 88.21	0.810	−0.06 (−0.40∼0.27)
17. During the past month, how much of the time have you felt confident?	67.67	64.38 ∼70.96	76.34	57.94 to 76.34	64.41	60.64∼68.19	76.34	57.94 to 76.34	0.276	−0.21 (−0.54∼0.11)
18. During the past month, how much of the time have you felt happy?	63.80	60.43∼67.17	73.27	54.88 to 73.27	62.74	59.26∼66.22	73.27	54.88 to 73.27	0.784	−0.07 (−0.39∼0.25)
19. During the past month, how much of the time have you felt able to do the things you want to do?	53.95	50.20∼57.71	65.32	46.92 to 65.32	52.58	48.66∼56.47	65.32	46.92 to 65.32	0.677	−0.08 (−0.40∼0.24)
20. During the past month, how much of the time have you felt eager to try new things?	62.56	58.86∼66.27	74.88	56.48 to 74.88	60.90	56.85∼64.94	74.88	41.22 to 74.88	0.738	−0.10 (−0.42∼0.22)

*Note:* All variables were performed the Wilcoxon rank test.

Abbreviations: IQR, interquartile range; Q, question; QIRC [37], quality of life impact of refractive correction.

## Data Availability

The relevant data supporting the research findings can be obtained from the corresponding author upon reasonable request.
